# Supplementary material for: Evaluation of Human Mesenchymal Stromal Cells as Carriers for the Delivery of Oncolytic HAdV-5 to Head and Neck Squamous Cell Carcinomas
Source: Viruses. 2023 Jan 13;15(1):218. doi: 10.3390/v15010218 (PMC9864513; doi:10.3390/v15010218)
Supplement: Supplementary file 1 [file viruses-15-00218-s001.zip › viruses-2103392-supplementary.pdf]

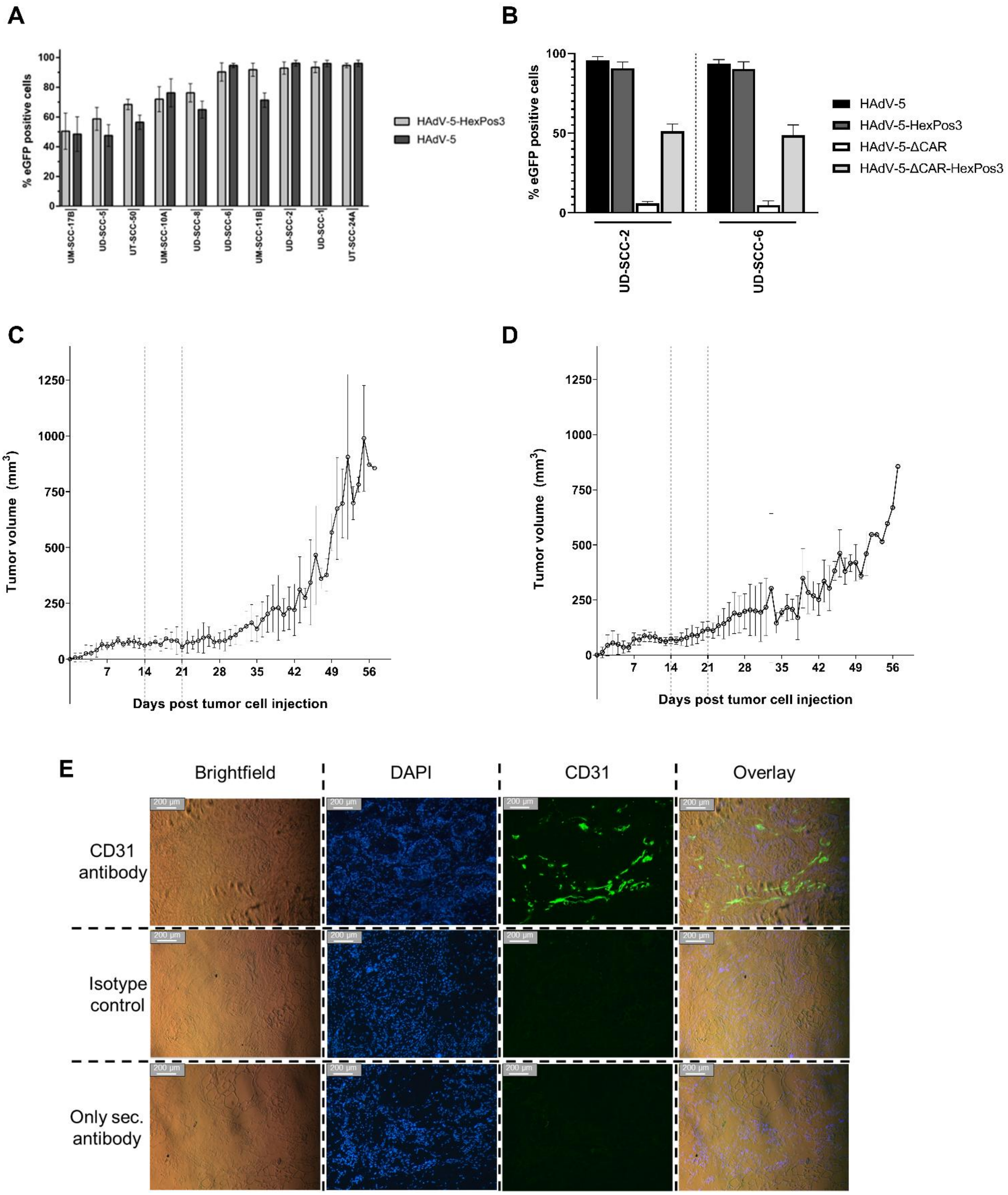


**Supplementary Figure S1** Establishment of a xenograft HNSCC model in NSG mice. (**A+B**) Cell lines derived from HNSCC were seeded into 96-well plates ($2 \times 10^4$ cells/well). The next day, cells were transduced with the indicated replication-incompetent first generation eGFP-expressing HAdV-5 vector (pMOI 1000). Enhanced GFP expression was analyzed 24 h later by flow cytometry. Results are shown as mean ± SD of biological triplicates. (**C+D**) $2 \times 10^6$ UD-SCC-2 (**C**) or UD-SCC-6 (**D**) cells were injected subcutaneously into the left flank of NSG mice. Tumor volumes were determined daily. Results are shown as mean ± standard deviation (UD-SCC-2: n = 6, UD-SCC-6: n = 7). (E) 6 µm cryosections were stained using a rat $\alpha$-mouse CD31 antibody and a secondary Alexa488-labeled goat anti-rat IgG antibody. ChromPure rat IgG was used as isotype control. Cell nuclei were stained with DAPI. A representative staining of a UD-SCC-2 xenograft tumor section is shown excised 39 days post tumor cell injection. Similar results were obtained for UD-SCC-6 xenograft tumor sections.

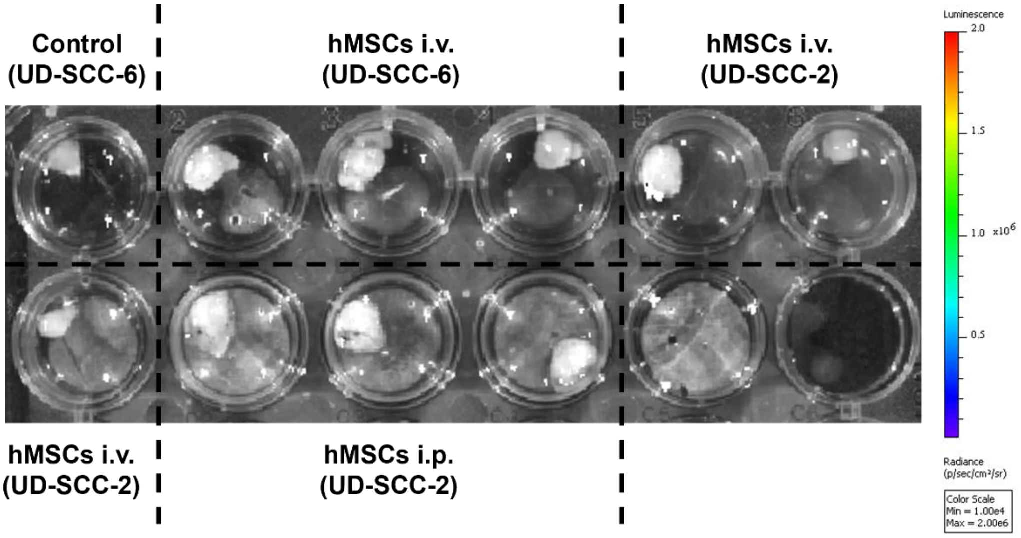


**Supplementary Figure S2** HNSCC tumors from mice injected with firefly luciferase-expressing hMSCs did not show intratumoral luciferase activity. 1 × $10^6$ BM-hMSCs (transduced 3 h before with a firefly luciferase-encoding replication-incompetent first generation HAdV-5 vector pre-incuabted with hFX) were injected i.v. or i.p. into tumor-bearing mice after 21 days of tumor growth. Seventy-two hours post-hMSC injection, the mice were sacrificed, and tumors were removed and transferred into PBS. The removed xenograft tumors were analyzed for luciferase activity using the IVIS 200 *in vivo* imaging system. Five minutes before analysis, firefly luciferase substrate was added to the PBS. A representative image from the IVIS 200 system is shown.
